# MicroRNA Expression in Salivary Supernatant of Patients with Pancreatic Cancer and Its Relationship with ZHENG

**DOI:** 10.1155/2014/756347

**Published:** 2014-07-14

**Authors:** Song Gao, Lian-Yu Chen, Peng Wang, Lu-Ming Liu, Zhen Chen

**Affiliations:** ^1^Department of Integrative Oncology, Fudan University Shanghai Cancer Center, Shanghai 200032, China; ^2^Department of Oncology, Shanghai Medical College, Fudan University, Shanghai 200032, China

## Abstract

In traditional Chinese medicine (TCM), diagnosis and prescriptions are based on the signs and symptoms which are recognized as ZHENG. The cornerstone of TCM is to differentially treat one ZHENG from others, which is also known as syndrome differentiation, and this relies on the gathering of clinical information through inspection, auscultation and olfaction, inquiry, and palpation. However, the biomolecular basis of the ZHENG remains unclear. In this study, the expressions of 384 cancer-related miRNAs in salivary supernatant of patients with pancreatic cancer were assessed by miRNA polymerase chain reaction (PCR) array, and the different expression patterns of miRNA in three different groups of ZHENG were studied with use of real-time quantitative PCR (qRT-PCR). Some miRNAs were found to be specifically expressed in some ZHENGs, for instance, miR-17, miR-21, and miR-181b in Shi-Re ZHENG and miR-196a in Pi-Xu ZHENG. This indicates that these miRNAs may play important roles in different ZHENG condition. Therefore, this study to some extent revealed the molecular basis of TCM ZHENG in pancreatic cancer.

## 1. Introduction

Traditional Chinese medicine (TCM), with a history of more than 3,000 years, emphasizes the comprehensive analysis of clinical information collected through four combined diagnostic methods: observation, auscultation and olfaction, inquiry, and palpation. The patients' disease condition will be classified into a specific ZHENG, which is the basis of prescription of Chinese herbs. ZHENG is the cornerstone in traditional Chinese medicine (TCM) theories. In cancer occurrence and its progression, a series of ZHENGs are involved. In recent years, many studies showed that TCM has certain advantages in the treatment of pancreatic cancer, while in most such studies it is difficult to interpret the advantages due to the lack of evidence-based medicine design. In TCM theory, the pathogenesis of pancreatic carcinoma may be the accumulation of dampness and heat, or the accumulation of dampness, heat, and toxicity. The related literature review indicated that Shi-Re, Pi-Xu, and Xue-Yu (dampness-heat, spleen-deficiency, and blood-stasis, resp.) are the top three types of TCM ZHENG in pancreatic cancer [1]. Accordingly, in this work, the expressions of some miRNAs related to pancreatic cancer (PC) under different ZHENG conditions were studied, and this work is expected to partially explore the molecular mechanism of TCM ZHENG in pancreatic cancer.

MicroRNA (miRNA), which is closely associated with the development of cancer, autoimmune disease, and other diseases, is single-stranded noncoding RNA in a variety of body fluids. During tumor development, aberrant expression of miRNAs can either inactivate tumor suppressor genes or activate oncogenes and thus promote tumor formation [2–8]. The expression of miRNAs is tissue-specific, detectable in blood [9], and correlated with clinical cancer development [10]. MicroRNA is highly stable in body fluids and can be easily detected. More and more scholars pay their attention to its expression in body fluids. Body fluids collection is convenient and trauma-free and thus can be easily accepted by patients, and there is no risk of hematogenous spread of diseases in sampling. Because detection of the expression levels of salivary miRNAs is convenient and noninvasive, it may be an option for early screening of pancreatic cancer. In conclusion, the role of salivary miRNAs is worthy of further study. This study focused on the specific expression of miRNA in the salivary supernatant in patients with pancreatic cancer, and the correlation between ZHENG and microRNA expression in pancreatic cancer was also investigated. Based on our results, the molecular basis of TCM ZHENG in pancreatic cancer is partially revealed, which may help making clinical syndrome differentiation and curative effect assessment for the pancreatic cancer more objective and standardized.

## 2. Materials and Methods

### 2.1. Patients

Thirty patients with newly diagnosed pancreatic cancer in Shanghai Cancer Center were enrolled in this study. Of all the patients (patient group), 17 were males and 13 were females, with age ranged from 33 to 78 years, and all were not treated before this study, that is, not receiving surgery, radiological, and interventional treatments. The diagnosis of pancreatic ductal carcinoma was confirmed by pathological results after surgery. Saliva from 32 healthy volunteers was collected as control (control group), and all volunteers had no history of cancer and family history of cancer. The two groups were comparable regarding sex and age (*P* > 0.05). The including criteria for patients were as follows. (1) The diagnosis was confirmed by tissue pathology. (2) All cases were newly diagnosed pancreatic cancer. The excluding criteria for patients were as follows. (1) Patients received surgery, radiological, and chemotherapeutic treatments before the study. (2) Patients were complicated with organic or functional heart, liver, kidney, and brain diseases.

### 2.2. Saliva Supernatant Collection

**Figure 1:**
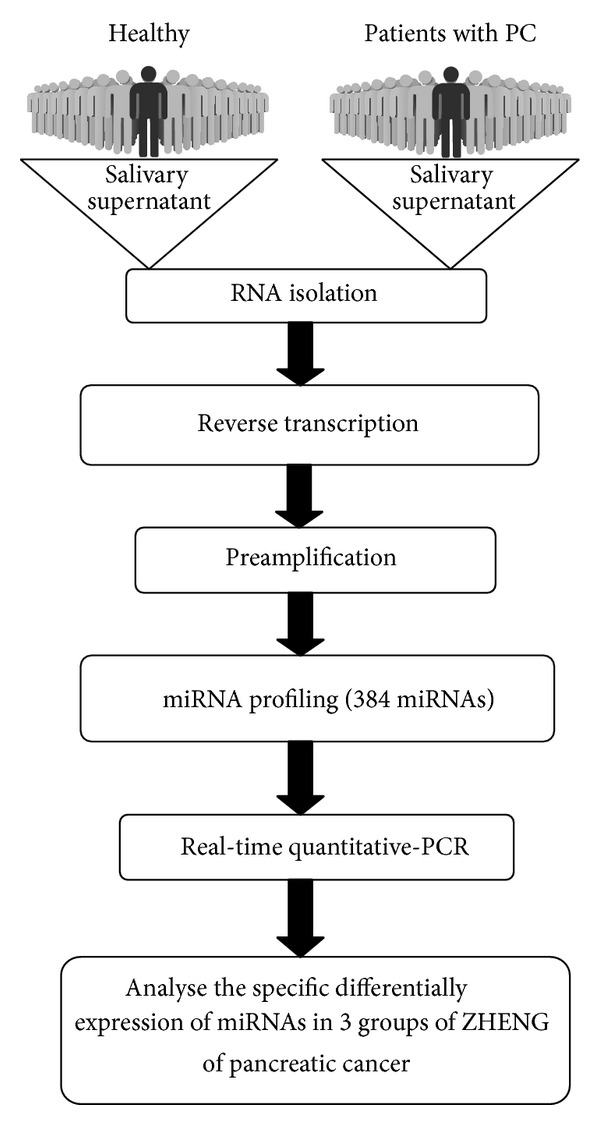
Work flow for processing salivary supernatant from 32 healthy volunteers and 30 PC patients and then screening 384 cancer-related miRNAs.

Saliva was centrifugated at 2500 g for 10 min at 4°C, and the supernatant was collected and centrifuged at 10,000 g for 1 min to remove remaining cells. The resultant supernatant was transferred into a new tube and stored at −80°C within 30 min of collection prior to RNA extraction (Figure 1).

### 2.3. RNA Isolation and Processing

The samples were centrifuged at 2500 g for 10 min, and only the supernatant was used for RNA extraction. Soluble miRNAs in supernatant were isolated with use of the miRNeasy Serum/Plasma Kit (Qiagen) according to the manufacturer's protocol. RNA was eluted in 14 uL of RNase-free water. cDNA was generated by miScript RTII kit (Qiagen) according to the manufacturer's recommendations. The prepared cDNA was diluted 1 : 4 in RNase-free water, 5 uL of the diluted cDNA was preamplified with the Qiagen miScript preAMP PCR kit, and then the preamplified cDNA was diluted 5-fold before qPCR detection.

### 2.4. Measurement of miRNAs

miRNA profiling was performed with use of miScript miRNA PCR array human miRNome (384-well plate) from Qiagen. For subsequent evaluations of candidate miRNAs, custom Qiagen 384-well plates with specific primer probes were used. A SYBR Green-based qPCR was performed with 2 uL cDNA in a 10 uL reaction volume in the Applied Biosystems 7900HT Fast Real-Time PCR System instrument, and the reaction conditions were as follows: 15 min at 95°C and 40 cycles of 15 s at 94°C, 30 s at 60°C, and 30 s at 72°C. All qPCR experiments were performed in 384-well plates, with a set of technical controls included on each plate (*Caenorhabditis elegans* miR-39 (C.el-miR-39), which was used as the spike-in control, and miRTC, which is a Qiagen proprietary synthetic oligonucleotide). To assess recoveries after RNA isolation, C.el-miR-39 was spiked into the sample before the extraction process. The efficiency of reverse transcription with miRTC was assessed, which was present in the nucleic acid mixture that was reverse-transcribed, preamplified, and detected by real-time qPCR.

### 2.5. miRNA Expression by Real-Time Quantitative Polymerase Chain Reaction

Selected microRNAs were measured using individual Qiagen microRNA arrays, with TaqMan reagents (RT mix and Universal Master Mix II). For saliva samples, Megaplex RT and preamplification were conducted to increase the limit of miRNA detection. RQ Manager 1.2 Software (ABI) was applied to generate Ct values. Relative miRNA levels were determined by ^ΔΔ^Ct using endogenous controls (miR-16). Ct values ≥ 35 were considered as negative amplification. Real-time PCR was performed using the miScript SYBR green PCR kit (Qiagen) according to the manufacturer's instructions. Five selective miRNA-specific RT primers were designed by primer premier 5.0 (Table 1 **)**. miR-16 were used as the endogenous normalizer to calculate the respective relative concentrations for the candidate miRNAs (Qiagen). Relative levels of miRNAs were assessed using the ^ΔΔ^Ct method.

### 2.6. Analysis of miRNAs in Salivary Supernatant and Statistical Analysis

The results from the qPCR reaction with SDS 2.4 software (Applied Biosystems) were obtained as threshold cycle values with automatic threshold and baseline values. All data were presented as mean ± standard deviation (SD). All statistics were performed with use of SPSS version 16.0. The results were then compared with use of one-way ANOVA with LSD among multiple groups or student *t*-test between two groups. Values of *P* < 0.05 were considered statistically significant in all tests.

## 3. Results

### 3.1. Candidate miRNAs Differentially Expressed in Patients with Pancreatic Cancer and in Healthy Controls

**Figure 2:**
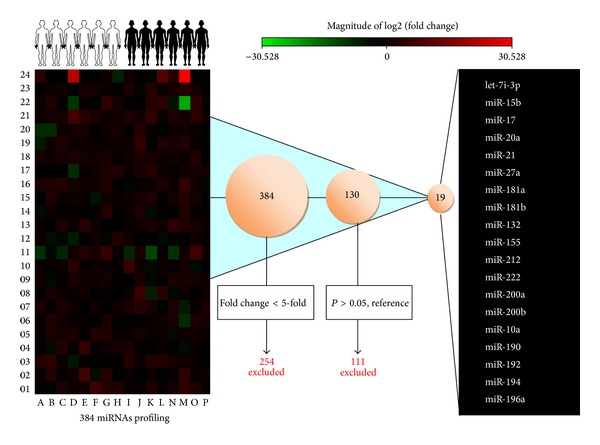
Heat map showing the fold changes in values of the 384 miRNAs in 32 healthy volunteers and 30 PC patients individually, compared with the mean of the healthy volunteers, a panel of the 19 selected miRNAs with proper melting curves, with miRNA values > 5-fold higher (*P* < 0.05) in PC patients than in healthy individuals.

An miRNA PCR array (miRBase version 18, containing 384 miRNAs; Qiagen) was used to detect the miRNAs fraction in salivary supernatant of 30 patients with pancreatic cancer and 32 healthy controls (Figure 1). Nineteen miRNAs were screened for the next phase of our study (Table 2). Custom qRT-PCR was performed to analyze the relative concentrations of the 19 candidate miRNAs in the saliva supernatant of the 30 patients and the 32 healthy volunteers (Figure 1). Of the 384 miRNAs investigated, 130 were upregulated more than 5-fold in patients with PC, among which 111 miRNAs were excluded since the difference was not significant (*P* > 0.01). Among the 19 eligible miRNAs (Figure 2), the top 5 miRNA candidates (miR-17, miR-21, miR-181a, miR-181b, and miR-196a) differentially expressed in the saliva samples of PC patients and healthy volunteers were chosen to be further confirmed in a larger cohort (based on miRWalk database https://www.ebi.ac.uk/arrayexpress/experiments/E-GEOD-53325/?query=saliva).

### 3.2. Relative Expression Levels of miR-17, miR-21, miR-181b, and miR-196a Were Different in Three ZHENGs of Pancreatic Cancer

**Figure 3:**
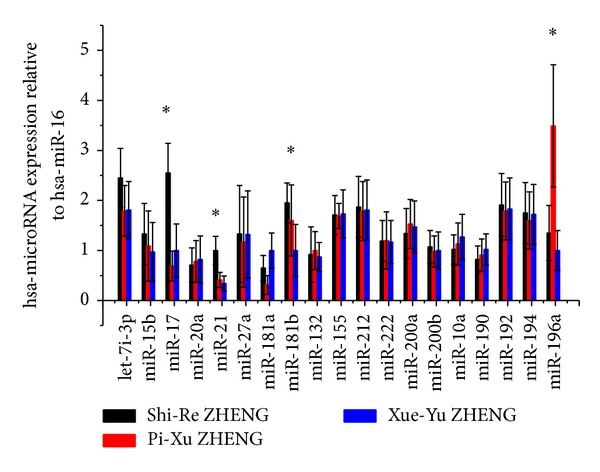
Comparison of differentially expression of 19 miRNAs among 3 ZHENGs of PC (Shi-Re ZHENG, Pi-Xu ZHENG, and Xue-Yu ZHENG) using the qRT-PCR; the bars indicate the fold change which was determined for the ratio of the expression of the PC salivary to the expression of the controls *P < 0.05.

The relative expression levels of miRNAs in 3 ZHENGs were studied, including Shi-Re ZHENG (*n* = 58), Pi-Xu ZHENG (*n* = 50), and Xue-Yu ZHENG (*n* = 45). Demographic data and clinical information of the participants were summarized in Table 3. The mean relative expression levels of miR-17, miR-21, and miR-181b in the saliva were significantly higher in patients with Shi-Re ZHENG than in patients with the other two ZHENGs (*P* < 0.05), whereas expression level of miR-196a was significantly higher in the Pi-Xu ZHENG group than in other two groups (*P* < 0.05) (Figure 3). The degree of upregulation of miR-196a was approximately 10-fold (*P* < 0.05) (Figure 3). The specific expressions of miR-17, miR-21, and miR-181b were able to distinguish Shi-Re ZHENG from the other two ZHENGs (*P* < 0.05), while Pi-Xu ZHENG was related to the higher expression of miR-196a. This may indicate the different molecular mechanisms of the syndromes in traditional Chinese medicine (ZHENG). The miRNA expression difference in the three different ZHENGs highlights the fact that these miRNAs play an important role, not only in the diseased human physiology but also in different ZHENG condition.

### 3.3. Inter- and Intra-Assay Imprecision

The entire experiment was performed with use of 384-well custom plates, each of which contained probes for the snoRNA/snRNA PCR Controls, C.el-miR-39 Primer, the miRTC oligonucleotide, and Positive PCR Control. All samples demonstrated good RNA recoveries (as measured by C.el-miR-39), with a mean intra-assay coefficient of variation (CV) of 7.2% and a mean interassay CV of 8.4% across all 153 samples and 2 plates. miRTC was used to measure the efficiency of reverse transcription. The mean intraplate CV (2.7%) and interplate CV (4.7%) indicated that the reverse-transcription reactions occurred at equivalent efficiencies across all samples. No template-control reactions and reactions without the reverse transcriptase were performed to determine whether the reverse transcription and PCR reactions worked properly. The melting temperatures of each miRNA were checked for all 153 samples to ensure the specificity of the probe and the mean (SD) melting temperature across all samples.

## 4. Discussion

In the clinical practice of traditional Chinese medicine (TCM), remedy prescription is made for different ZHENG (also known as syndrome), which is the objective classification of the patients' disease condition. ZHENG is kind of classification based on experience according to the patients conditions, and the prescription of herbs is made for different syndromes of TCM. ZHENG is also a kind of pathology of the disease development of a body in a certain stage. It reflects the nature of pathological change at a certain stage and reveals the intrinsic quality of disease more completely, profoundly, and accurately than symptoms [23]. However, the biomolecular basis of the ZHENG remains unclear. In recent years, TCM has shown certain advantages in treating pancreatic cancer, while the results from most of these studies are difficult to be widely applied due to the lack of enough evidence-based medicine arguments as well as few studies on TCM ZHENG of pancreatic cancer. Compared with biomolecular science and Western medicine, ZHENG in TCM is short of objectivity, accuracy, and reproducibility. Since their discovery in human plasma, extracellular miRNAs have been explored as biomarkers of cancer. Method for miRNA detection in peripheral blood has been well developed, and it is accurate and stable. The value of miRNA as a diagnostic marker has been confirmed due to its highly stable expression in peripheral blood and close association with tumor pathological process, tumor source, and particular functions [10–12, 14].

Several studies have found stable miRNA profiles in bodily fluids, such as saliva, which is a complex liquid that comprises secretions from the major and minor salivary glands. There is also an extensive blood supply to these glands. Molecules present in plasma are also present in saliva, such as DNA, RNA, and proteins [24]. Therefore, tumor-related miRNAs may similarly exist in saliva. There are emerging studies which have demonstrated that cancer-derived exosome-like microvesicles are capable of activating transcription in salivary gland cells and altering the salivary gland cell-derived exosome-like microvesicles [25]. The finding indicated that tumor-derived exosomes could function as the shuttle between the distal tumor and the oral cavity leading to the development of discriminatory salivary biomarkers. In general, the salivary tumor-related miRNAs may come from the blood supplying to salivary glands or the cancer-derived exosome-like microvesicles, which need further studies to be confirmed.

Saliva is a mixture of proteins, DNA, RNA, fatty acids, and a variety of microbes, and it can be the feasible sample for the diagnosis of a variety of diseases. Researchers from Scripps Research Institute of University of Rochester, UCLA, and UCSF analyzed the protein components from parotid gland, submaxillary gland, and sublingual gland secretions and finally identified a total of 1116 different proteins [26]. They compared these proteins with proteins in blood and tears and finally found some proteins in saliva, matched with proteins in blood, which play a role in Alzheimer's disease, breast cancer, and diabetes. The proteins studied in this work can be used to construct a proteomic map of saliva and also can be used as the biomarkers for PC diagnosis.

In recent years, there are tremendous works of body fluids for the diagnosis of disease. MicroRNA in saliva, as a new diagnostic marker, is also a hot topic. Park et al. [27] reported 50 kinds of miRNAs in the supernatants of saliva from patients with oral squamous cell carcinoma. Further study showed that levels of miR-125a and miR-200a were significantly lower in saliva of patients than in that of healthy subjects. Liu et al. [28] found that expression of miR-31 in supernatant of body fluids from OSCC patients was significantly higher than in that of healthy subjects, and the expression level decreased after tumor resection, which indicates that miRNAs could be used in the diagnosis and prognosis of OSCC. At the same time, researchers have isolated miRNAs from saliva of healthy people and patients with Sjogren's syndrome [29]. Similarly, there could be some miRNAs in saliva, which can reflect the tumorigenesis, development, and metastasis, as well as prognosis of pancreatic cancer. Our study aims to find the new pancreatic cancer biomarkers in saliva by screening differentially expressed miRNAs in pancreatic cancer patients and healthy population and further to identify the specific miRNAs particularly expressed in different ZHENGs of pancreatic cancer. To our knowledge, there were no such studies described the salivary miRNome in the clinical setting of ZHENG before this study.

In this work, we isolated and analyzed 384 miRNAs in the salivary supernatant from patients with PC and healthy controls; 19 miRNAs were identified to be differentially expressed between patients with PC (*n* = 30) and healthy controls (*n* = 32). This is in agreement with previously published microRNA profiling studies, most of which have shown that microRNA expression in serum or tumor samples seems globally higher than in normal samples [11–13, 15–22]. Interestingly, the differentially expressed miRNAs described here (including let-7, miR-21, and miR-181a) are similar to those previously published microRNA profiling studies. However, some of the differentially expressed microRNAs described in this study are not consistent with the reported study [30]. This discrepancy could be due to the patient individual differences, RNA degradation due to salivary amylase, and the influence of salivary collection method. Our results indicate that differentially expressed miRNAs may play an important role in development and progression of PC by deregulating specific target oncogenes or tumor suppressors. Furthermore, the same miRNA may play opposite roles in tumor pathogenesis, that is, as an oncogene in certain cancers and as a tumor suppressor in others [31]. Therefore, targets of most of the miRNAs including their pathways and functions still remain to be studied urgently. The sample size for our microarray analysis is relatively small. However, this is not like those studies of mRNA gene marker screening, which may need large sample size to obtain reliable profile. In this work, a limited number of microRNAs were studied.

Nineteen miRNAs were chosen for the further analysis in another 153 patients. There were significant differences in the expression of 4 microRNAs (miR-17, miR-21, miR-181b, and miR-196a) in three patient groups with different ZHENG. These results suggest that microRNAs may play an important role in the molecular pathogenesis of PC and could be included as one of the indicators for distinguishing the syndromes of traditional Chinese medicine (ZHENG). No such data was reported concerning the miR-17 miR-21, miR-181b, and miR-196a and their relevance to clinicopathologic behavior and syndromes of traditional Chinese medicine in patients with PC previously.

However, some studies have demonstrated a link between these 4 miRNAs and the prognosis in pancreatic cancer. MiR-17-5p is reported to be overexpressed in pancreatic cancer, and it plays an important role in carcinogenesis and cancer progression [32]. Serum exosomal miR-17 was higher in PC patients than in non-PC patients and healthy participants. High levels of miR-17 were significantly correlated with metastasis and advanced stage of PC [33]. The former studies have identified miR-21 as overexpressed in early pancreatic cancer lesions, pancreatic tumors, and pancreatic cancer-derived cell lines [34]. miR-21 is one of the most cited miRNAs and has emerged as the miRNA most frequently associated with poor outcome in cancer [35]. MiR-21 and miR-196a were overexpressed in pancreatic carcinomas compared to benign aspirates [36]. Some study suggested that the expression of miR-181b was higher in cell lines of pancreatic cancer, such as BxPC3, Panc1, and PSN1 cells [37]. In brief, these 4 miRNAs may play a role of oncogene in pancreatic cancer and it may become a new early diagnosis mark and therapy target of pancreatic cancer. Unlike miR-17 and miR-21, which are all deregulated in the same direction in a variety of cancers [13, 14], members of the miR-181 family are upregulated in some cancers and downregulated in others [38–41]. It has been reported that miR-181 downregulates the homeobox protein Hox-A11, which acts as a suppressor in differentiation process, and thus a functional link between miR-181 family and the complicated process of differentiation was established. Based on this, the discrepancies could also be due to microRNA target differences in different tumor differentiation stage and microenvironment. However, the precise targets of miRNA in patients with various ZHENGs remain unclear at present, and how miRNA is involved in tumor formation and affects the prognosis also remains unclear.

This study mainly focuses on the investigation of searching for the correlation between the expression of miRNAs in saliva and the syndromes. At the same time, we hoped to find the discriminatory salivary biomarkers, which could be readily detected upon the development of pancreatic cancer. The main purpose of this paper is exploring the relationship between miRNAs in salivary supernatant and the syndromes, making clinical syndrome differentiation and curative effect assessment for the pancreatic cancer more objective and standardized.

## 5. Conclusions

In summary, miRNAs in salivary supernatant of PC patients were studied and 4 miRNAs were identified, that is, miR-17, miR-21, miR-181b, and miR-196a, as sensitive indicators for treatment based on syndrome differentiation. However, there are some limitations in this work. Firstly, the sample size of this study is relatively small, and therefore, to confirm the results, further study with larger sample size is required. Secondly, although salivary supernatant from healthy people is used as control in gene profiling analysis, oral foreign body, such as food debris, may contaminate the results by changing the ingredient and percentage of miRNA. This may contribute to controversial reports on miRNA expression in cancer. Thirdly, the precise roles of these 4 miRNAs (miR-17, miR-21, miR-181b, and miR-196a) and their target mRNAs regulation in PC progression remain unclear. Further in vitro and in vivo studies should be performed to enrich the knowledge of this nascent field. Nevertheless, our results indicate that these 4 miRNAs may be good candidate molecular markers in ZHENG differentiation.

## Figures and Tables

**Table 1:** RT primers for amplification of the human mature miRNAs used in validation of microRNA microarray results.

Name	RT primer	Product length (bp)
miR-16	F primer 5′-CGCGCTAGCAGCACGTAAATA-3′	**71**
	R primer 5′-GTGCAGGGTCCGAGGT-3′	
	Probe 5′-TTCGCACTGGATACGACCGCCAA-3′	

miR-17	F primer 5′-CGGCGGCAAAGTGCTTACAG-3′	**73**
	R primer 5′-GTGCAGGGTCCGAGGT-3′	
	Probe 5′-TTCGCACTGGATACGACCTACCTGCA-3′	

miR-21	F primer 5′-GCGGCGGCTAGCTTATCAGAC-3′	**74**
	R primer 5′-GTGCAGGGTCCGAGGT-3′	
	Probe 5′-TTCGCACTGGATACGACTCAACATCAG-3′	

miR-181a	F primer 5′-GCCCGAACATTCAACGCTGT-3′	**72**
	R primer 5′-GTGCAGGGTCCGAGGT-3′	
	Probe 5′-TTCGCACTGGATACGACACTCACCG-3′	

miR-181b	F primer 5′-CGCGCAACATTCATTGCTGT-3′	**66**
	R primer 5′-GTGCAGGGTCCGAGGT-3′	
	Probe 5′-TTCGCACTGGATACGACACCCAC-3′	

miR-196a	F primer 5′-CGGCTTTGGCACTAGCACATT-3′	**71**
	R primer 5′-GTGCAGGGTCCGAGGT-3′	
	Probe 5′-TTTGCGCACTGGATACGACAGCAA-3′	

**Table 2:** The expression level of pancreatic cancer related miRNAs in salivary supernatant.

miRNA	Function	The relative quantitative expression	Fold change	Reference
let-7i-3p	Antioncogene	Down	67.96	[11]
miR-15b	Antioncogene	Down	77.73	[12]
miR-17	Oncogene	Up	425.26	[13]
miR-20a	Oncogene	Up	5.19	[13]
miR-21	Oncogene	Up	388.16	[14]
miR-27a	Oncogene	Up	5.65	[15]
miR-181a	Oncogene	Up	156.43	[16]
miR-181b	Oncogene	Up	362.49	[16]
miR-132	Oncogene	Up	9.415	[17]
miR-155	Oncogene	Up	9.56	[18]
miR-212	Oncogene	Up	11.87	[17]
miR-222	Oncogene	Up	6.38	[16]
miR-200a	Oncogene	Up	10.7	[19]
miR-200b	Oncogene	Up	5.39	[12]
miR-10a	Oncogene	Up	32.82	[20]
miR-190	Oncogene	Up	6.63	[12]
miR-192	Oncogene	Up	6.22	[21]
miR-194	Oncogene	Up	18.2	[22]
miR-196a	Oncogene	Up	6.14	[16]

**Table 3:** Baseline patient characteristics in the 3 groups of ZHENG associated with pancreatic cancer.

Clinical features	153 patients
Sex	
Male	76 (49.7%)
Female	77 (50.3%)
Median age (years)	58.8
Three TCM ZHENGs	
Shi-Re (dampness-heat)	58 (37.9%)
Pi-Xu (spleen-deficiency)	50 (32.7%)
Xue-Yu (blood-stasis)	45 (29.4%)
Treatment	
Cycle of TCM-based therapy	≥2
Median duration (day)	62 (54.1–69.9)
Response	
Disease progression	74 (49.7%)
Grade 3-4 toxicity	36 (24.6%)
Alternative treatment	43 (25.6%)
